# Poly[(dimethyl­formamide)(μ_4_-2,2′-sulfanediyldibenzoato)nickel(II)]

**DOI:** 10.1107/S1600536810007749

**Published:** 2010-03-06

**Authors:** Jiang-Bo Xie

**Affiliations:** a College of Materials Science and Engineering, North University of China, Taiyuan, Shanxi, 030051, People’s Republic of China

## Abstract

The title centrosymmetric dinuclear Ni^II^ complex, [Ni(C_14_H_8_O_4_S)(C_3_H_7_NO)]_*n*_, was prepared *via* reaction of Ni(NO_3_)_2_·6H_2_O and thio­salicylic acid, with H_2_O and dimethyl­formamide (DMF) as the mixed solvent. The central Ni^II^ ion is five-coordinated by five O atoms from DMF and from the carboxyl­ate groups of the organic ligand. The symmetry-related coordination polyhedra inter­link into centrosymmetric dimeric units and these, in turn, are linked into infinite chains propagating parallel to [100].

## Related literature

For high-dimensional coordination polymers, see: Li *et al.* (2009 ▶, 2010 ▶).
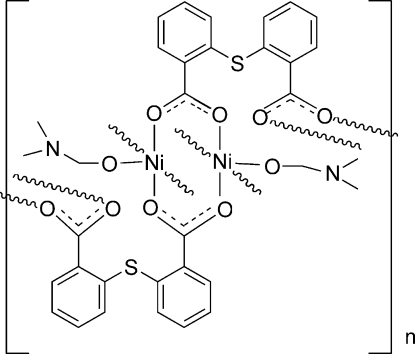


## Experimental

### 

#### Crystal data

[Ni(C_14_H_8_O_4_S)(C_3_H_7_NO)]
*M* *_r_* = 808.12
Triclinic, 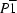

*a* = 8.5196 (2) Å
*b* = 10.5240 (2) Å
*c* = 11.0138 (3) Å
α = 67.241 (1)°
β = 79.0410 (11)°
γ = 71.796 (1)°
*V* = 862.33 (3) Å^3^
*Z* = 1
Mo *K*α radiation
μ = 1.27 mm^−1^
*T* = 298 K
0.30 × 0.25 × 0.19 mm

#### Data collection

Bruker APEXII area-detector diffractometer
Absorption correction: multi-scan (*SADABS*; Sheldrick, 2004 ▶) *T*_min_ = 0.701, *T*_max_ = 0.794
11190 measured reflections
3350 independent reflections
2553 reflections with *I* > 2σ(*I*)
*R*_int_ = 0.035

#### Refinement

*R*[*F* ^2^ > 2σ(*F* ^2^)] = 0.036
*wR*(*F* ^2^) = 0.079
*S* = 1.01
3350 reflections
228 parameters
H-atom parameters constrained
Δρ_max_ = 0.44 e Å^−3^
Δρ_min_ = −0.35 e Å^−3^

### 

Data collection: *APEX2* (Bruker, 2004 ▶); cell refinement: *SAINT* (Bruker, 2004 ▶); data reduction: *SAINT*; program(s) used to solve structure: *SHELXS97* (Sheldrick, 2008 ▶); program(s) used to refine structure: *SHELXL97* (Sheldrick, 2008 ▶); molecular graphics: *XP* in *SHELXTL* (Sheldrick, 2008 ▶); software used to prepare material for publication: *SHELXL97*.

## Supplementary Material

Crystal structure: contains datablocks I, global. DOI: 10.1107/S1600536810007749/bg2334sup1.cif

Structure factors: contains datablocks I. DOI: 10.1107/S1600536810007749/bg2334Isup2.hkl

Additional supplementary materials:  crystallographic information; 3D view; checkCIF report

## Figures and Tables

**Fig. 1.**
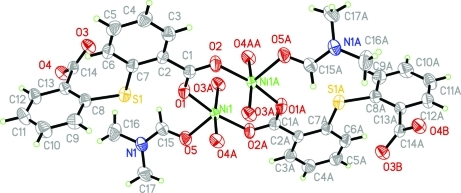
Displacement ellipsoid plot (40% probability level) of the title compound(I), with atom numbering of structurally unique non-H.

**Fig. 2.**
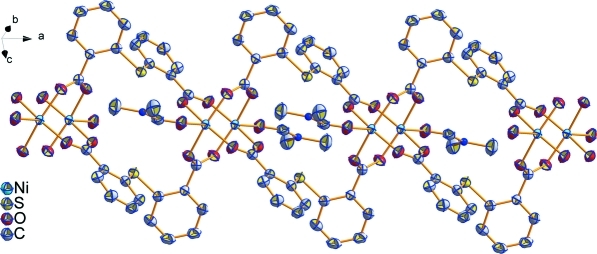
The packing diagram of the title compound (I).

## supplementary crystallographic information

### Comment

Organic carboxylates as ligands attract much attention not only because of versatile coordination modes but also owing to their ability to facilitate the formation of high-dimensional coordination polymers (Li *et al.*, 2009; Li *et al.*, 2010). One such example, namely, thiosalicylic acid, is a semi-rigid, multidentate ligand that can provide up to four donor atoms with variable coordination modes. Unlike the imidazole-4,5-dicarboxylic acid and benzimidazole-5,6-dicarboxylic acid with nitrogen and oxygen coordinated dots, the thiosalicylic acid only has oxygen coordinated dot. So it can form low-dimensional compound. This is therefore considered as an excellent candidate for generating one-dimensional compound with chain structure.

In the title complex, the Ni^II^ atom is coordinated by one oxygen atom from one DMF ligand and four oxygen atoms from the thiosalicylic acid carboxylates, giving a centrosymmetric dimeric structure with a Ni···Ni distance of 2.6374 (7) Å (Fig. 1). A one-dimensional infinite chain is formed due to the bidentate bridging mode shown by all the thiosalicylic acid carboxylates (Fig. 2). The Ni—O bond distances vary from 1.947 (2) Å to 2.129 (2) Å. The O—Ni—O angles are in the range of 88.16 (9) to 168.24 (8) °. As far as we know, examples of dinuclear Ni^II^ complex bridged by thiosalicylic acid and DMF have not been characterized so far.

### Experimental

A solution obtained by dissolving 0.145 g (0.5 mmol) Ni(NO_3_)_2_.6H_2_O in 4 ml DMF and 10 ml H_2_O was added. The mixture was stirred until complete dissolution. To the stirred solution was added equimolar quantities 0.136 g (0.5 mmol) thiosalicylic acid. The green solution was then under 160 °C for 72 h in a 23 ml Teflon-lined stainless-steel autoclave. Afterthe reaction, the bomb was cooled to room temperature in a rate of 5 °C per hour. Green prismatic crystals were collected and dried in air. Yield: *ca.*82 % on the basis of Ni.

### Refinement

All H atoms were positioned in calculated positions, with C—H distances of 0.93 and 0.96 Å,and with Uiso~(H) = 1.2 or 1.5 Ueq~(C).

### Crystal data

**Table:**

[Ni(C_14_H_8_O_4_S)(C_3_H_7_NO)]	*Z* = 1
*M**_r_* = 808.12	*F*(000) = 416
Triclinic, *P*1	*D*_x_ = 1.556 Mg m^−^^3^
Hall symbol: -P 1	Mo *K*α radiation, λ = 0.71073 Å
*a* = 8.5196 (2) Å	Cell parameters from 2701 reflections
*b* = 10.5240 (2) Å	θ = 2.4–22.3°
*c* = 11.0138 (3) Å	µ = 1.27 mm^−^^1^
α = 67.241 (1)°	*T* = 298 K
β = 79.0410 (11)°	Block, green
γ = 71.796 (1)°	0.30 × 0.25 × 0.19 mm
*V* = 862.33 (3) Å^3^	

### Data collection

**Table:**

Bruker APEXII area-detector diffractometer	3350 independent reflections
Radiation source: fine-focus sealed tube	2553 reflections with *I* > 2σ(*I*)
graphite	*R*_int_ = 0.035
φ and ω scan	θ_max_ = 26.0°, θ_min_ = 2.0°
Absorption correction: multi-scan (*SADABS*; Sheldrick, 2004)	*h* = −9→10
*T*_min_ = 0.701, *T*_max_ = 0.794	*k* = −12→12
11190 measured reflections	*l* = −13→13

### Refinement

**Table:**

Refinement on *F*^2^	Primary atom site location: structure-invariant direct methods
Least-squares matrix: full	Secondary atom site location: difference Fourier map
*R*[*F*^2^ > 2σ(*F*^2^)] = 0.036	Hydrogen site location: inferred from neighbouring sites
*wR*(*F*^2^) = 0.079	H-atom parameters constrained
*S* = 1.01	*w* = 1/[σ^2^(*F*_o_^2^) + (0.0295*P*)^2^ + 0.4*P*] where *P* = (*F*_o_^2^ + 2*F*_c_^2^)/3
3350 reflections	(Δ/σ)_max_ = 0.001
228 parameters	Δρ_max_ = 0.44 e Å^−^^3^
0 restraints	Δρ_min_ = −0.34 e Å^−^^3^

### Special details

**Table:**

Geometry. All esds (except the esd in the dihedral angle between two l.s. planes) are estimated using the full covariance matrix. The cell esds are taken into account individually in the estimation of esds in distances, angles and torsion angles; correlations between esds in cell parameters are only used when they are defined by crystal symmetry. An approximate (isotropic) treatment of cell esds is used for estimating esds involving l.s. planes.
Refinement. Refinement of *F*^2^ against ALL reflections. The weighted *R*-factor wR and goodness of fit *S* are based on *F*^2^, conventional *R*-factors *R* are based on *F*, with *F* set to zero for negative *F*^2^. The threshold expression of *F*^2^ > σ(*F*^2^) is used only for calculating *R*-factors(gt) etc. and is not relevant to the choice of reflections for refinement. *R*-factors based on *F*^2^ are statistically about twice as large as those based on *F*, and *R*- factors based on ALL data will be even larger.

### Fractional atomic coordinates and isotropic or equivalent isotropic displacement parameters (Å^2^)

**Table:**

	*x*	*y*	*z*	*U*_iso_*/*U*_eq_	
Ni1	0.37381 (4)	0.57945 (3)	0.55076 (3)	0.03249 (12)	
S1	−0.02998 (9)	0.75102 (9)	0.29878 (7)	0.0481 (2)	
O1	0.2572 (2)	0.5904 (2)	0.40641 (19)	0.0499 (5)	
O2	0.4743 (3)	0.4593 (2)	0.3200 (2)	0.0551 (6)	
O3	−0.3145 (3)	0.5998 (2)	0.33626 (19)	0.0501 (5)	
O4	−0.5286 (2)	0.7322 (2)	0.4242 (2)	0.0521 (5)	
O5	0.1675 (3)	0.7125 (2)	0.6255 (2)	0.0547 (6)	
N1	−0.1056 (3)	0.8241 (3)	0.6247 (3)	0.0535 (7)	
C1	0.3275 (4)	0.5360 (3)	0.3199 (3)	0.0417 (7)	
C2	0.2315 (3)	0.5674 (3)	0.2057 (3)	0.0381 (6)	
C3	0.3089 (4)	0.5029 (3)	0.1132 (3)	0.0501 (8)	
H3	0.4126	0.4380	0.1281	0.060*	
C4	0.2369 (4)	0.5317 (4)	0.0004 (3)	0.0561 (8)	
H4	0.2922	0.4894	−0.0614	0.067*	
C5	0.0810 (4)	0.6248 (3)	−0.0189 (3)	0.0553 (8)	
H5	0.0308	0.6456	−0.0948	0.066*	
C6	−0.0010 (4)	0.6872 (3)	0.0722 (3)	0.0494 (8)	
H6	−0.1072	0.7477	0.0582	0.059*	
C7	0.0723 (3)	0.6616 (3)	0.1858 (3)	0.0411 (7)	
C8	−0.2076 (4)	0.8749 (3)	0.2161 (3)	0.0429 (7)	
C9	−0.1898 (4)	1.0066 (3)	0.1271 (3)	0.0571 (9)	
H9	−0.0855	1.0235	0.1077	0.069*	
C10	−0.3225 (5)	1.1122 (4)	0.0670 (3)	0.0665 (10)	
H10	−0.3077	1.1993	0.0069	0.080*	
C11	−0.4766 (4)	1.0887 (3)	0.0962 (3)	0.0620 (9)	
H11	−0.5668	1.1595	0.0550	0.074*	
C12	−0.4984 (4)	0.9601 (3)	0.1866 (3)	0.0529 (8)	
H12	−0.6039	0.9455	0.2069	0.063*	
C13	−0.3648 (3)	0.8520 (3)	0.2478 (3)	0.0388 (7)	
C14	−0.4029 (4)	0.7160 (3)	0.3450 (3)	0.0399 (7)	
C15	0.0244 (4)	0.7207 (3)	0.6097 (3)	0.0472 (7)	
H15	0.0070	0.6507	0.5859	0.057*	
C16	−0.2706 (4)	0.8325 (4)	0.5980 (4)	0.0821 (12)	
H16A	−0.2647	0.7545	0.5709	0.123*	
H16B	−0.3115	0.9213	0.5289	0.123*	
H16C	−0.3440	0.8271	0.6765	0.123*	
C17	−0.0867 (4)	0.9353 (4)	0.6631 (4)	0.0730 (11)	
H17A	0.0279	0.9356	0.6498	0.110*	
H17B	−0.1239	0.9177	0.7546	0.110*	
H17C	−0.1515	1.0264	0.6103	0.110*	

### Atomic displacement parameters (Å^2^)

**Table:**

	*U*^11^	*U*^22^	*U*^33^	*U*^12^	*U*^13^	*U*^23^
Ni1	0.0266 (2)	0.0328 (2)	0.0312 (2)	−0.00384 (15)	−0.00182 (14)	−0.00739 (15)
S1	0.0385 (5)	0.0590 (5)	0.0440 (4)	−0.0096 (4)	−0.0055 (3)	−0.0165 (4)
O1	0.0380 (12)	0.0645 (14)	0.0418 (12)	−0.0035 (11)	−0.0075 (9)	−0.0189 (11)
O2	0.0390 (13)	0.0620 (14)	0.0583 (14)	0.0012 (11)	−0.0136 (10)	−0.0217 (11)
O3	0.0488 (13)	0.0383 (12)	0.0507 (13)	−0.0109 (10)	0.0077 (10)	−0.0083 (10)
O4	0.0458 (13)	0.0440 (12)	0.0561 (13)	−0.0135 (10)	0.0116 (11)	−0.0125 (10)
O5	0.0396 (13)	0.0615 (14)	0.0619 (14)	−0.0011 (11)	−0.0042 (11)	−0.0302 (12)
N1	0.0392 (16)	0.0548 (17)	0.0596 (17)	−0.0016 (13)	−0.0001 (13)	−0.0230 (14)
C1	0.0382 (18)	0.0402 (17)	0.0422 (17)	−0.0145 (15)	−0.0034 (14)	−0.0062 (14)
C2	0.0373 (17)	0.0401 (16)	0.0344 (15)	−0.0146 (14)	−0.0054 (12)	−0.0058 (13)
C3	0.0479 (19)	0.0488 (19)	0.0492 (19)	−0.0120 (16)	−0.0056 (15)	−0.0124 (16)
C4	0.059 (2)	0.066 (2)	0.0479 (19)	−0.0171 (19)	−0.0054 (16)	−0.0247 (17)
C5	0.064 (2)	0.061 (2)	0.0452 (18)	−0.0191 (19)	−0.0137 (16)	−0.0157 (17)
C6	0.0444 (19)	0.056 (2)	0.0467 (18)	−0.0127 (16)	−0.0133 (15)	−0.0122 (16)
C7	0.0380 (17)	0.0432 (17)	0.0398 (16)	−0.0177 (14)	−0.0025 (13)	−0.0068 (13)
C8	0.0430 (18)	0.0410 (17)	0.0387 (16)	−0.0096 (14)	−0.0039 (13)	−0.0084 (14)
C9	0.056 (2)	0.055 (2)	0.055 (2)	−0.0266 (18)	0.0023 (17)	−0.0072 (17)
C10	0.075 (3)	0.044 (2)	0.060 (2)	−0.018 (2)	−0.001 (2)	0.0035 (17)
C11	0.059 (2)	0.0440 (19)	0.057 (2)	0.0006 (17)	−0.0058 (18)	−0.0013 (16)
C12	0.0407 (19)	0.0497 (19)	0.055 (2)	−0.0071 (16)	−0.0006 (15)	−0.0090 (16)
C13	0.0368 (17)	0.0370 (16)	0.0371 (16)	−0.0094 (14)	0.0002 (13)	−0.0088 (13)
C14	0.0338 (17)	0.0419 (18)	0.0397 (16)	−0.0105 (14)	−0.0036 (13)	−0.0090 (14)
C15	0.048 (2)	0.0482 (19)	0.0381 (17)	−0.0065 (16)	0.0032 (15)	−0.0155 (15)
C16	0.045 (2)	0.111 (3)	0.092 (3)	−0.013 (2)	−0.004 (2)	−0.044 (3)
C17	0.060 (2)	0.064 (2)	0.094 (3)	−0.0011 (19)	0.003 (2)	−0.042 (2)

### Geometric parameters (Å, °)

**Table:**

Ni1—O2^i^	1.947 (2)	C5—C6	1.373 (4)
Ni1—O4^ii^	1.9695 (19)	C5—H5	0.9300
Ni1—O3^iii^	1.9751 (19)	C6—C7	1.400 (4)
Ni1—O1	1.9790 (19)	C6—H6	0.9300
Ni1—O5	2.129 (2)	C8—C9	1.389 (4)
Ni1—Ni1^i^	2.6374 (6)	C8—C13	1.390 (4)
S1—C7	1.781 (3)	C9—C10	1.371 (4)
S1—C8	1.786 (3)	C9—H9	0.9300
O1—C1	1.259 (3)	C10—C11	1.368 (5)
O2—C1	1.258 (3)	C10—H10	0.9300
O3—C14	1.249 (3)	C11—C12	1.379 (4)
O4—C14	1.258 (3)	C11—H11	0.9300
O5—C15	1.236 (3)	C12—C13	1.391 (4)
N1—C15	1.325 (4)	C12—H12	0.9300
N1—C17	1.450 (4)	C13—C14	1.507 (4)
N1—C16	1.460 (4)	C15—H15	0.9300
C1—C2	1.504 (4)	C16—H16A	0.9600
C2—C3	1.391 (4)	C16—H16B	0.9600
C2—C7	1.405 (4)	C16—H16C	0.9600
C3—C4	1.375 (4)	C17—H17A	0.9600
C3—H3	0.9300	C17—H17B	0.9600
C4—C5	1.379 (4)	C17—H17C	0.9600
C4—H4	0.9300		
			
O2^i^—Ni1—O4^ii^	90.48 (9)	C7—C6—H6	119.4
O2^i^—Ni1—O3^iii^	88.17 (9)	C6—C7—C2	118.1 (3)
O4^ii^—Ni1—O3^iii^	168.24 (8)	C6—C7—S1	120.9 (2)
O2^i^—Ni1—O1	168.07 (8)	C2—C7—S1	121.0 (2)
O4^ii^—Ni1—O1	89.02 (9)	C9—C8—C13	118.9 (3)
O3^iii^—Ni1—O1	89.89 (8)	C9—C8—S1	117.6 (2)
O2^i^—Ni1—O5	97.16 (8)	C13—C8—S1	123.1 (2)
O4^ii^—Ni1—O5	97.19 (8)	C10—C9—C8	121.5 (3)
O3^iii^—Ni1—O5	94.56 (8)	C10—C9—H9	119.2
O1—Ni1—O5	94.73 (8)	C8—C9—H9	119.2
O2^i^—Ni1—Ni1^i^	84.58 (6)	C11—C10—C9	119.6 (3)
O4^ii^—Ni1—Ni1^i^	81.54 (6)	C11—C10—H10	120.2
O3^iii^—Ni1—Ni1^i^	86.70 (6)	C9—C10—H10	120.2
O1—Ni1—Ni1^i^	83.56 (6)	C10—C11—C12	120.1 (3)
O5—Ni1—Ni1^i^	177.88 (6)	C10—C11—H11	120.0
C7—S1—C8	102.75 (13)	C12—C11—H11	120.0
C1—O1—Ni1	123.11 (19)	C11—C12—C13	120.9 (3)
C1—O2—Ni1^i^	123.63 (19)	C11—C12—H12	119.6
C14—O3—Ni1^iii^	119.78 (18)	C13—C12—H12	119.6
C14—O4—Ni1^iv^	125.92 (19)	C8—C13—C12	119.0 (3)
C15—O5—Ni1	120.6 (2)	C8—C13—C14	124.6 (3)
C15—N1—C17	120.9 (3)	C12—C13—C14	116.4 (2)
C15—N1—C16	120.9 (3)	O3—C14—O4	126.0 (3)
C17—N1—C16	118.2 (3)	O3—C14—C13	118.5 (2)
O2—C1—O1	124.9 (3)	O4—C14—C13	115.4 (3)
O2—C1—C2	117.1 (3)	O5—C15—N1	123.4 (3)
O1—C1—C2	118.0 (3)	O5—C15—H15	118.3
C3—C2—C7	119.1 (3)	N1—C15—H15	118.3
C3—C2—C1	117.4 (3)	N1—C16—H16A	109.5
C7—C2—C1	123.5 (3)	N1—C16—H16B	109.5
C4—C3—C2	122.1 (3)	H16A—C16—H16B	109.5
C4—C3—H3	118.9	N1—C16—H16C	109.5
C2—C3—H3	118.9	H16A—C16—H16C	109.5
C3—C4—C5	118.5 (3)	H16B—C16—H16C	109.5
C3—C4—H4	120.7	N1—C17—H17A	109.5
C5—C4—H4	120.7	N1—C17—H17B	109.5
C6—C5—C4	120.9 (3)	H17A—C17—H17B	109.5
C6—C5—H5	119.6	N1—C17—H17C	109.5
C4—C5—H5	119.6	H17A—C17—H17C	109.5
C5—C6—C7	121.2 (3)	H17B—C17—H17C	109.5
C5—C6—H6	119.4		

Symmetry codes: (i) −*x*+1, −*y*+1, −*z*+1; (ii) *x*+1, *y*, *z*; (iii) −*x*, −*y*+1, −*z*+1; (iv) *x*−1, *y*, *z*.
